# Supplementary material for: Meaningful changes in motor function in Duchenne muscular dystrophy (DMD): A multi-center study
Source: PLoS One. 2024 Jul 10;19(7):e0304984. doi: 10.1371/journal.pone.0304984 (PMC11236155; doi:10.1371/journal.pone.0304984)
Supplement: S1 Text — (DOCX) [file pone.0304984.s001.docx]

### S1 Text. Supplementary methods

For an outcome measure, Y, measured at a baseline visit, t_0_, and a follow-up visit, t_1_, a change between these visits (Y_t1_ - Y_t0_), may be considered to reflect true improvement with q x 100% confidence if the lower limit of a one-sided q x 100% confidence interval for the change is greater than 0:

This condition can be expressed by:

(Y_t1_ - Y_t0_) - (z_q_ * SE(Y_t1_ - Y_t0_)) > 0

Or equivalently,

Y_t1_ - Y_t0_ > (z_q_ * SE(Y_t1_ - Y_t0_))

SE(Y_t1_ - Y_t0_) = sqrt(Var(Y_t1_ - Y_t0_))

Var (Y_t1_ - Y_t0_) = Var (Y_t1_) + Var (Y_t0_) - 2 Cov (Y_t1_, Y_t0_)

Assuming autocorrelation is non-negative, which is a reasonable assumption for functional performance outcomes.

Var (Y_t1_ - Y_t0_) <= Var (Y_t1_) + Var (Y_t0_)

We further assume that variance around an individual's true trajectory is constant over time, so that Var(Y_t1_) = Var(Y_t0_) and that this quantity is estimated by RSE^2.

Var (Y_t1_ - Y_t0_) <= 2 Var (Y_t0_) = 2*(RSE^2)

Therefore,
SE (Y_t1_ - Y_t0_) = sqrt(Var(Y_t1_ - Y_t0_)) <= sqrt(2*RSE^2) = sqrt(2)*RSE

Therefore Y_t1_ - Y_t0_ indicates true improvement if

Y_t1_ - Y_t0_ > z_q_* sqrt(2)*RSE = MDC

For 80% confidence, z_0.8_ = 0.84, MDC = 0.84 * sqrt (2) * RSE = 1.2* RSE

For 90% confidence, z_0.9_ = 1.28, MDC = 1.28 * sqrt (2) * RSE = 1.8* RSE

Arguing similarly, as above Y_t1_ - Y_t0_ indicates true worsening if:

Y_t1_ - Y_t0_ < z_q_* sqrt(2)*RSE = MDC

For 80% confidence in true worsening, MDC = - (0.84 * sqrt (2) * RSE) = - 1.2* RSE

For 90% confidence in true worsening, MDC = - (1.28 * sqrt (2) * RSE) = - 1.8* RSE
