# Supplementary material for: Meaningful changes in motor function in Duchenne muscular dystrophy (DMD): A multi-center study
Source: PLoS One. 2024 Jul 10;19(7):e0304984. doi: 10.1371/journal.pone.0304984 (PMC11236155; doi:10.1371/journal.pone.0304984)
Supplement: S1 Table — (DOCX) [file pone.0304984.s002.docx]

**S1 Table. Description of clinical trial data sources**

|  | **Tadalafil DMD trial placebo arm** | **Ataluren phase 3 trial (ACT-DMD)**  **placebo arm** | **Ataluren phase 2b trial placebo arm** | **Drisapersen phase 3 trial (DEMAND III) placebo arm** | **Drisapersen phase 2 trial placebo arm (NCT01153932)** | **Drisapersen phase 2 trial placebo arm (NCT01462292)** | **Marathon 001** | **Marathon 002** |
| --- | --- | --- | --- | --- | --- | --- | --- | --- |
| Data source type | Placebo arm data from a multi-center, randomized, double-blind, placebo-controlled phase 3 trial | Placebo arm data from a multi-center, randomized, double-blind, placebo-controlled phase 3 trial | Placebo arm data from a multi-center, randomized, double-blind, placebo-controlled phase 2b trial | Placebo arm data from a multi-center, randomized, double-blind, placebo-controlled phase 3 trial | Placebo arm data from a multi-center, exploratory, randomized, double-blind, placebo-controlled phase 2 trial | Placebo arm data from a multi-center, exploratory, randomized, double-blind, placebo-controlled phase 2 trial | Data from a multi-center, randomized, double-blind, placebo-controlled phase 3 trial | Data from a multi-center, randomized, double-blind, placebo-controlled phase 3 trial |
| Study identifier | NCT01865084 | NCT01826487 | NCT00592553 | NCT01254019 | NCT01153932 | NCT01462292 | - | - |
| Study locations | 63 sites in 15 countries | 54 sites in 18 countries | 37 sites in 11 countries | 44 centers in 19 countries | 13 centers in 9 countries | 13 centers in the US | 9 centers in 2 countries (the US, Canada) | 5 centers in Italy |
| Data collection time period (for data used in this study) | 2013 - 2015 | 2013- 2014 | 2008 - 2009 | 2010 - 2013 | 2010 - 2012 | 2011 - 2013 | 1993 – 1995 | 1988 – 1991 |
| Key inclusion & exclusion  criteria | - Proven DMD based on diagnostic criteria - Age 7- 14 years - 6MWD between 200 and 400 meters, 2 baseline 6MWDs differing by <20%, - Systemic corticosteroid therapy for ≥6 months with a stable regimen ≥3 months. - Left ventricular ejection fraction ≥50% | - Nonsense mutation DMD, confirmed by gene sequencing - Age 7-16 years - 6MWD ≥150 meters - Systemic corticosteroid use for ≥6 months and no substantial change to dose or dosing regimen and regimen for ≥3 months prior to start of treatment, and expectation that this would not change during the study with the expectation of remaining on similar dose/regimen for the study duration | - Documented nonsense mutation DMD - Age ≥5 years   6MWD ≥75 meters | - DMD resulting from an exon 51 skipping amenable mutation - - Age ≥5 years - 3 pre-treatment 6MWD tests ≥75 meters within 20% of each other; - Glucocorticosteroid therapy for ≥6 months and stable dose and regimen for ≥3 months prior to screening, with the expectation of remaining on similar dose/regimen for the study duration | - Confirmed drisapersen-correctable mutation - Age ≥5 years - 2 pre-treatment 6MWD tests ≥75 meters within 20% of each other - Glucocorticosteroid therapy for ≥6 months and stable dose and regimen for ≥3 months prior to screening | - DMD resulting from an exon 51 skipping amenable mutation - Age ≥5 years - 6MWD ≥75 meters - Glucocorticosteroid therapy for ≥6 months and stable dose and regimen for ≥3 months prior to screening, with the exception of weight adjustments | - Proven DMD based on diagnostic criteria - Age 5-15 years - Included patients who were non-ambulatory at baseline (n=45) - Excluded patients with long-term use of oral GC, or regular recent use steroids, and any use of steroids in the immediate past | - Proven DMD based on diagnostic criteria - Age 5-11 years - Ambulatory patients only - No exclusion criteria relating to steroid use |
| Typical standard of care, including glucocorticoid use and physical therapy | -50% of patients on deflazacort; 50% on prednisolone at baseline  -73% received daily steroids | 47% of patients on deflazacort; 53% on prednisolone at baseline  -74% received daily steroids | 70% of patients were receiving glucocorticoids at baseline | -43% of patients on deflazacort; 57% on prednisolone at baseline  -54% received daily steroids | -At baseline, 61% were on continuous corticosteroid regimen; 39% were on an intermittent regimen | 94% of patients on continuous corticosteroid regimen; 6% on intermittent corticosteroid regimen at baseline | In this trial, patients were randomized to deflazacort 0.9 mg/kg/day (n=51), deflazacort 1.2 mg/kg/day (n=49), prednisone 0.75 mg/kg/day (n=46) or placebo (n=50) | In this trial, patients were randomized to deflazacort (n=20) or placebo (n=11) |
| Number of patients in trial arm * | 116 | 115 | 57 | 61 | 18 | 16 | 196 | 29 |

*Reflects number of patients in trial arm, not all of whom were eligible or had available data for use in MDC analyses. Sample sizes used in MDC analyses are provided in S3 Table.
