# Supplementary material for: Meaningful changes in motor function in Duchenne muscular dystrophy (DMD): A multi-center study
Source: PLoS One. 2024 Jul 10;19(7):e0304984. doi: 10.1371/journal.pone.0304984 (PMC11236155; doi:10.1371/journal.pone.0304984)
Supplement: S2 Table — (DOCX) [file pone.0304984.s003.docx]

**S2 Table. Description of real-world and natural history data sources**

|  | **Real-world data and natural history data sources** | | | | | |
| --- | --- | --- | --- | --- | --- | --- |
|  | **PRO-DMD-01** | **Leuven** | **iMDEX** | **North Star UK** | **CCHMC** | **ImagingDMD** |
| Data source type | Prospectively collected natural history data | Curated RWD from boys with DMD from routine clinical practice at the Universitaire Ziekenhuizen pediatric neurology clinic in Leuven, Belgium | Prospective, longitudinal, multicenter observational study at neuromuscular centers | Prospective natural history study from specialist neuromuscular centers in the United Kingdom | Curated clinical data from electronic health records of boys with DMD from the Comprehensive Neuromuscular Center at CCHMC | A longitudinal, multicenter, observational study of boys with DMD and age-matched controls without DMD (not used in this study) |
| Study identifier | NCT01753804 | - | NCT02780492 | - | - | NCT01484678 |
| Study locations | 16 sites across USA, South America, and Europe | 1 center in Belgium | 5 centers in Europe | 24 centers in the United Kingdom | 1 center in Cincinnati, USA | Recruited from across the United States but received assessments at 3 study centers in |
| Data collection time period (for data used in this study) | 2012 - 2016 | 2007 - present | 2012 - 2019 | 2003 - 2015 | 2004 - 2017 | –2010-2016 |
| Key inclusion & exclusion  criteria | - Genetically proven DMD - Age 3 to 18 years - Willing and able to comply with protocol requirements - Life expectancy of at least 3 years - Able to give informed assent and/or consent in writing signed by the subject and/or parent(s)/legal guardian | - Genetically proven DMD - Aged 4.5 to 17.5 years - No severe cognitive or behavioral disorder impairing compliance^a^ | - Diagnosis of DMD documented by MLPA or a standard genetic test for the disorder, genotypically confirmed to have an out-of-frame deletion(s) that could be corrected by skipping exon 51 or 53 or 45 or 44 or 46 or 50 or 52 - Ambulant children from 5 years old and teenagers with DMD - Ability to walk independently for at least 75 meters in 6 minutes at recruitment - Standard of care for DMD as recommended by the NorthStar UK and TREAT-NMD (i.e.: on glucocorticoids treatment) - Sufficiently preserved pulmonary function (FVC >30%) and absence of symptoms of cardiac failure | - DMD diagnosis confirmed by genetic testing and/or a muscle biopsy | - DMD diagnosis confirmed by genetic testing and/or muscle biopsy | - Ambulatory and nonambulatory boys - between 5 and 18 y of age - Diagnosed with DMD - Onset of symptoms before age 5 y - No contraindication to an MRI examination - No unstable medical problems - Must be able to cooperate during testing and not have cognitive deficits - No secondary conditions that may affect muscle metabolism, muscle - function, or functional ability |
| Typical standard of care, including glucocorticoid use and physical therapy | At baseline 208 subjects (78%) were using steroids for DMD, mainly in a continuous (56.2%) or intermittent (15.4%) regimen, and 59 (22.1%) used none (mostly younger boys). | - Glucocorticoid usually prescribed from age of 4 to 6 years onwards; 90% received 0.90 mg/kg daily deflazacort - Physical therapy advice for prevention of contractures | - Standard of care for DMD as recommended by the NorthStar UK and TREAT-NMD (i.e.: on glucocorticoids treatment)  - 67% received daily steroids | - All patients included were treated according to the standards of care, comprising therapy with GC administered either as daily prednisolone/deflazacort or intermittently (ie, prednisolone 10 days on:10 days off or alternate days)  - 50% received daily steroids | - Standard care including prescribing glucocorticoid steroids usually starting from age 4 years and onwards - Stretching exercise and AFO uses for prevention of contractures - Nutritional consultation for weight management and ensure good calcium and Vit D intakes | Majority of patients are  current glucocorticoid users: 74% of enrollees received  steroids at study entry; 87% received steroids at any time during course of study.  Most participants do not receive care at the ImagingDMD sites. Differences in standard of  care are expected both for glucocorticoids and physical therapy. |
| Number of patients in database* | 269 | 150 | 90 | 533 | 600 | 95 |

*Reflects number of patients in database, not all of whom were eligible or had available data for use in MDC analyses. Sample sizes used in MDC analyses are provided in S3 Table.
