# Supplementary material for: Meaningful changes in motor function in Duchenne muscular dystrophy (DMD): A multi-center study
Source: PLoS One. 2024 Jul 10;19(7):e0304984. doi: 10.1371/journal.pone.0304984 (PMC11236155; doi:10.1371/journal.pone.0304984)
Supplement: S3 Table — (DOCX) [file pone.0304984.s004.docx]

**S3 Table. Sample size, median follow-up and mean age at first visit in MDC analyses**

|  | **NSAA analyses** | | | | **6MWD analyses** | | | | **4SC analyses** | | | |
| --- | --- | --- | --- | --- | --- | --- | --- | --- | --- | --- | --- | --- |
|  | **Number of assessments** | **Number of patients** | **Median follow-up (months)** | **Age at first visit (mean [SD])** | **Number of assessments** | **Number of patients** | **Median follow-up (months)** | **Age at first visit (mean [SD])** | **Number of assessments** | **Number of patients** | **Median follow-up (months)** | **Age at first visit (mean [SD])** |
| **All patients** | **5917** | **1012** | **24.7** | **8.1 (2.3)** | **3701** | **625** | **11.6** | **8.8 (2.1)** | **6402** | **1029** | **19.3** | **8.3 (2.3)** |
| **RWD/NHD sources** | **4319** | **743** | **35.6** | **7.8 (2.4)** | **1566** | **275** | **29.6** | **8.6 (2.4)** | **3966** | **604** | **36.2** | **8.0 (2.5)** |
| CCHMC | 1558 | 264 | 45.7 | 7.8 (2.6) | - | - | - | - | 1737 | 283 | 45.1 | 7.6 (2.5) |
| Leuven | 244 | 38 | 36.4 | 8.5 (2.7) | 489 | 50 | 44.2 | 8.4 (2.1) | 935 | 70 | 71.7 | 7.7 (2.0) |
| iMDEX | 142 | 27 | 30.3 | 7.8 (2.1) | 74 | 17 | 24.1 | 8.9 (2.3) | 144 | 27 | 30.8 | 7.7 (2.1) |
| NSUK | 1576 | 256 | 38.0 | 7.3 (2.1) | - | - | - | - | - | - | - | - |
| PRO-DMD-01 | 799 | 158 | 24.1 | 8.6 (2.4) | 798 | 159 | 23.9 | 8.6 (2.5) | 874 | 165 | 24.6 | 8.6 (2.6) |
| Imaging DMD | - | - | - | - | 205 | 49 | 36.6 | 8.5 (2.2) | 276 | 59 | 48.1 | 8.4 (2.2) |
| **Clinical trial Arms** | **1598** | **269** | **11.1** | **9.0 (1.8)** | **2135** | **350** | **11.0** | **9.1 (1.9)** | **2436** | **425** | **11.0** | **8.8 (2.0)** |
| Tadalafil DMD trial placebo | 583 | 102 | 11.5 | 9.4 (1.7) | 636 | 112 | 11.5 | 9.4 (1.8) | 611 | 106 | 11.5 | 9.4 (1.8) |
| Marathon 001 | - | - | - | - | - | - | - | - | 449 | 106 | 10.3 | 8.1 (2.1) |
| Marathon 002 | - | - | - | - | - | - | - | - | 26 | 7 | 28.6 | 8.3 (0.9) |
| Ataluren phase 2b placebo | - | - | - | - | 344 | 40 | 11.0 | 9.1 (2.2) | 338 | 38 | 11.0 | 9.1 (2.2) |
| ACT-DMD placebo | 687 | 100 | 11.0 | 9.3 (1.5) | 724 | 110 | 11.0 | 9.4 (1.7) | 686 | 101 | 11.0 | 9.3 (1.5) |
| DEMAND III placebo | 246 | 50 | 11.0 | 8.0 (1.9) | 270 | 55 | 11.0 | 8.4 (2.3) | 244 | 50 | 11.0 | 8.1 (2.2) |
| Drisapersen phase 2 placebo (NCT01153932) | 82 | 17 | 11.1 | 7.3 (1.2) | 82 | 17 | 11.1 | 7.3 (1.2) | 82 | 17 | 11.1 | 7.3 (1.2) |
| Drisapersen phase 2 placebo (NCT01462292) | - | - | - | - | 79 | 16 | 11.0 | 8.4 (1.7) | - | - | - | - |

Note: ‘-‘ indicates that the outcome measure was not available in the data source.
