# Supplementary material for: Meaningful changes in motor function in Duchenne muscular dystrophy (DMD): A multi-center study
Source: PLoS One. 2024 Jul 10;19(7):e0304984. doi: 10.1371/journal.pone.0304984 (PMC11236155; doi:10.1371/journal.pone.0304984)
Supplement: S4 Table — (DOCX) [file pone.0304984.s005.docx]

**S4 Table. North Star Ambulatory Assessment details**

| Clinical trial placebo arms |
| --- |
| **Tadalafil DMD trial:** NSAA was a secondary endpoint in this trial and was measured every 12 weeks. Evaluators were trained therapists/physiotherapists. |
| **ACT-DMD:** NSAA was an exploratory endpoint in this trial and was measured every 8 weeks. NSAA was evaluated using standardized procedures included in a study manual. Videotaping of assessments was planned at each visit for outcome measure monitoring and quality control. |
| **DEMAND III:** NSAA was a secondary endpoint in this trial and was measured every 12 weeks. NSAA was evaluated using a standardized manual within the study procedures manual with specific instructions for grading. Video snaps were used in the training program to ensure evaluator reliability. Site staff members were trained on functional efficacy endpoints by highly experienced and well-trained physiotherapists, who provided refresher training as required throughout the study. |
| **Drisapersen phase 2 placebo (NCT01153932):** NSAA was a secondary endpoint in this trial and was measured every 12 weeks. NSAA was evaluated using a standardized manual within the study procedures manual with specific instructions for grading. Video snaps were used in the training program to ensure evaluator reliability |
| Real-world and natural history data sources |
| **PRO-DMD-01:** NSAA was assessed every 6 months. Based on the study protocol, functional assessments in general were to be performed by clinical evaluators with experience in the handling of patients with DMD. Clinical evaluators were to be trained in the use of all equipment and test procedures to ensure reliability. Ideally, the same evaluator was to test the same participant on each occasion. To avoid bias, the clinical evaluators were not to review any previous testing results. For NSAA specifically, the clinical evaluator manual included for each item, details on child’s starting position, instruction to be given to child, description of scoring detail and example photographs for different score levels. |
| **Leuven:** NSAA was assessed approximately every 6 months during multidisciplinary follow up clinics. The clinical evaluator was the same as for the clinical trials at the center. The evaluator has been trained in the use of equipment and test procedures and participated in the DEMAND, Tadalafil and ACT DMD trials. |
| **iMDEX:** NSAA was assessed approximately every 6 months. NSAA evaluations were done based on the clinical evaluator manual for the measure. |
| **North Star UK:** NSAA was assessed approximately every 6 months. A training program and standard operating procedures existed across all the participating sites to ensure standardization of procedures. Clinical and physiotherapy assessments were recorded in forms specifically developed for that purpose and centrally collected for analysis. |
| **CCHMC:** NSAA was assessed approximately every 6 to 12 months. The NSAA evaluations were part of the motor evaluations by the clinic physical therapists during the patients’ multidisciplinary clinic visits. These physical therapists were also clinical evaluators for the DMD clinical trials and received training with the initiation of each DMD clinical trial. |
