# Supplementary material for: Meaningful changes in motor function in Duchenne muscular dystrophy (DMD): A multi-center study
Source: PLoS One. 2024 Jul 10;19(7):e0304984. doi: 10.1371/journal.pone.0304984 (PMC11236155; doi:10.1371/journal.pone.0304984)
Supplement: S5 Table — (DOCX) [file pone.0304984.s006.docx]

**S5 Table. 6MWD assessment details**

| Clinical trial placebo arms |
| --- |
| **Tadalafil DMD trial:** 6MWD was the primary endpoint in this this trial and was assessed using modified American Thoracic Society (ATS) criteria. 6MWD assessments were done at baseline and every 12 weeks during the 48-week trial period. |
| **Ataluren phase 2b trial:** 6MWD was the primary endpoint in this trial and was assessed using modified American Thoracic Society (ATS) criteria. 6MWD assessments were done at baseline and every 6 weeks during the 48-week trial period. |
| **ACT-DMD :** 6MWD was the primary endpoint in this trial and was assessed using modified American Thoracic Society (ATS) criteria. 6MWD assessments were done at baseline and every 8 weeks during the 48-week trial period. |
| **DEMAND III:** 6MWD was the primary endpoint in this trial and was assessed using modified American Thoracic Society (ATS) criteria. 6MWD assessments were done at baseline and every 12 weeks during the 48-week trial period. |
| **Drisapersen phase 2 trials:** 6MWD was the primary endpoint in these trials and was assessed using modified American Thoracic Society (ATS). 6MWD assessments were done at baseline and every 12 weeks during the trial periods. |
| Real-world and natural history data sources |
| **PRO-DMD-01:** 6MWD was assessed using modified ATS criteria. Testing guidelines were provided to each center in operations manual. There were two evaluators for each test. 6MWD was done every 6 months. |
| **UZ Leuven:** 6MWD was assessed using modified ATS criteria by the same two trained and experienced physiotherapists at the site, approximately every 6 months. |
| **iMDEX:** 6MWD was assessed using modified ATS criteria and was assessed approximately every 6 months. All centers in the study had a formal training on its execution. |
| **ImagingDMD:** 6MWD was assessed using modified ATS criteria. Evaluators at each site were trained and certified. Assessments were performed annually (more frequently for a subset of patients in the first year of follow-up). |
