# Supplementary material for: Meaningful changes in motor function in Duchenne muscular dystrophy (DMD): A multi-center study
Source: PLoS One. 2024 Jul 10;19(7):e0304984. doi: 10.1371/journal.pone.0304984 (PMC11236155; doi:10.1371/journal.pone.0304984)
Supplement: S6 Table — (DOCX) [file pone.0304984.s007.docx]

**S6 Table. Magnitude of change in NSAA total required to have 80% or 90% confidence that true change has occurred, among all patients, by data source, and by subgroups of function and age**

|  | MDC  (80% confidence) | MDC  (90% confidence) |
| --- | --- | --- |
| All patients | 2.78 | 4.17 |
| By data source |  |  |
| RWD/NHD | 2.77 | 4.15 |
| CCHMC | 2.58 | 3.87 |
| Leuven | 2.52 | 3.79 |
| iMDEX | 3.16 | 4.74 |
| NSUK | 2.99 | 4.49 |
| PRO-DMD-01 | 2.64 | 3.96 |
| Clinical Trial Arms | 2.80 | 4.20 |
| Tadalafil DMD trial placebo | 2.86 | 4.29 |
| ACT-DMD placebo | 2.79 | 4.19 |
| DEMAND III placebo | 2.73 | 4.10 |
| Drisapersen phase 2 placebo  (NCT01153932) | 2.68 | 4.02 |
| By baseline NSAA total score |  |  |
| <21 | 3.00 | 4.50 |
| 21-28 | 2.69 | 4.04 |
| >28 | 2.50 | 3.76 |
| By age group, years |  |  |
| ≤7 | 2.66 | 3.99 |
| 7-12 | 2.80 | 4.19 |
| >12 | 2.80 | 4.20 |
