# Supplementary material for: Meaningful changes in motor function in Duchenne muscular dystrophy (DMD): A multi-center study
Source: PLoS One. 2024 Jul 10;19(7):e0304984. doi: 10.1371/journal.pone.0304984 (PMC11236155; doi:10.1371/journal.pone.0304984)
Supplement: S7 Table — (DOCX) [file pone.0304984.s008.docx]

**S7 Table. Magnitude of change in 6MWD (meters) required to have 80% or 90% confidence that true change has occurred, among all patients, by data source, and by subgroups of function and age**

|  | MDC  (80% confidence) | MDC  (90% confidence) |
| --- | --- | --- |
| All patients | 36.3 | 54.5 |
| By data source |  |  |
| RWD/NHD | 39.1 | 58.6 |
| **Leuven** | 41.5 | 62.3 |
| iMDEX | 35.0 | 52.5 |
| PRO-DMD-01 | 38.5 | 57.7 |
| ImagingDMD | 36.6 | 55.0 |
| Clinical trial arms | 34.2 | 51.3 |
| **Tadalafil DMD trial placebo** | 35.6 | 53.8 |
| Ataluren phase 2b placebo | 33.6 | 50.4 |
| ACT-DMD placebo | 34.1 | 51.1 |
| DEMAND III placebo | 32.7 | 49.0 |
| Drisapersen phase 2 placebo (NCT01153932) | 34.8 | 52.2 |
| Drisapersen phase 2 placebo (NCT01462292) | 30.5 | 45.8 |
| By baseline 6MWD, meters |  |  |
| 76-200 | 60.0 | 90.0 |
| 201-400 | 33.9 | 50.8 |
| >400 | 34.2 | 51.2 |
| By age group, years |  |  |
| ≤7 | 43.4 | 65.1 |
| 7-12 | 35.6 | 53.4 |
| >12 | 35.6 | 53.5 |
