# Supplementary material for: Meaningful changes in motor function in Duchenne muscular dystrophy (DMD): A multi-center study
Source: PLoS One. 2024 Jul 10;19(7):e0304984. doi: 10.1371/journal.pone.0304984 (PMC11236155; doi:10.1371/journal.pone.0304984)
Supplement: S8 Table — (DOCX) [file pone.0304984.s009.docx]

**S8 Table. Magnitude of change in 4SC time (seconds, with completion times truncated at 12 seconds) and 4SC velocity (stairs/second) required to have 80% or 90% confidence that true change has occurred, among all patients, by data source, and by subgroups of function and age**

|  | 4SC time (seconds) | | 4SC velocity (stairs/second) | |
| --- | --- | --- | --- | --- |
|  | MDC  (80% confidence) | MDC  (90% confidence) | MDC  (80% confidence) | MDC  (90% confidence) |
| All patients | 1.30 | 1.96 | 0.35 | 0.52 |
| By data source |  |  |  |  |
| RWD/NHD | 1.30 | 1.95 | 0.36 | 0.54 |
| **CCHMC** | 1.05 | 1.58 | 0.37 | 0.55 |
| Leuven | 1.55 | 2.32 | 0.39 | 0.58 |
| iMDEX | 1.90 | 2.85 | 0.42 | 0.63 |
| PRO-DMD-01 | 1.37 | 2.06 | 0.33 | 0.49 |
| ImagingDMD | 1.21 | 1.81 | 0.27 | 0.40 |
| Clinical trial arms | 1.31 | 1.97 | 0.33 | 0.49 |
| Tadalafil DMD trial placebo | 1.46 | 2.19 | 0.26 | 0.39 |
| Marathon 001 | 1.14 | 1.71 | 0.33 | 0.49 |
| Marathon 002 | 1.29 | 1.94 | 0.27 | 0.41 |
| Ataluren phase 2b placebo | 1.26 | 1.90 | 0.31 | 0.46 |
| ACT-DMD placebo | 1.28 | 1.91 | 0.39 | 0.59 |
| DEMAND III placebo | 1.40 | 2.10 | 0.31 | 0.47 |
| Drisapersen phase 2 placebo (NCT01153932) | 1.21 | 1.81 | 0.31 | 0.46 |
| By baseline 4SC completion time [velocity] |  |  |  |  |
| <2s [> 2 stairs/s] | 0.90 | 1.35 | 0.53 | 0.79 |
| 2 to 8s [0.5 to 2 stairs/s] | 1.19 | 1.79 | 0.31 | 0.46 |
| >8s [< 0.5 stairs/s] | 1.85 | 2.78 | 0.25 | 0.38 |
| By age group (years) |  |  |  |  |
| ≤7 | 1.06 | 1.58 | 0.37 | 0.55 |
| 7-12 | 1.32 | 1.99 | 0.34 | 0.52 |
| >12 | 1.39 | 2.08 | 0.35 | 0.53 |
