# Supplementary material for: Meaningful changes in motor function in Duchenne muscular dystrophy (DMD): A multi-center study
Source: PLoS One. 2024 Jul 10;19(7):e0304984. doi: 10.1371/journal.pone.0304984 (PMC11236155; doi:10.1371/journal.pone.0304984)
Supplement: S9 Table — (DOCX) [file pone.0304984.s010.docx]

**S9 Table. Magnitude of change in 4SC time (seconds, with completion times truncated at 30 seconds) and 4SC velocity (stairs/second) required to have 80% or 90% confidence that true change has occurred, among all patients, by data source, and by subgroups of function and age**

|  | 4SC time (seconds) | | 4SC velocity (stairs/second) | |
| --- | --- | --- | --- | --- |
|  | MDC  (80% confidence) | MDC  (90% confidence) | MDC  (80% confidence) | MDC  (90% confidence) |
| All patients | 3.21 | 4.81 | 0.35 | 0.53 |
| By data source |  |  |  |  |
| RWD/NHD | 3.25 | 4.87 | 0.36 | 0.55 |
| CCHMC | 1.81 | 2.72 | 0.37 | 0.55 |
| Leuven | 4.09 | 6.14 | 0.39 | 0.59 |
| iMDEX | 4.97 | 7.45 | 0.44 | 0.66 |
| PRO-DMD-01 | 3.87 | 5.80 | 0.33 | 0.49 |
| ImagingDMD | 3.68 | 5.52 | 0.27 | 0.41 |
| Trial Placebo Arms | 3.15 | 4.72 | 0.33 | 0.50 |
| Tadalafil Placebo Arm | 3.67 | 5.51 | 0.27 | 0.40 |
| Marathon 001 | 2.04 | 3.06 | 0.33 | 0.49 |
| Marathon 002 | 1.86 | 2.78 | 0.27 | 0.41 |
| Ataluren phase 2b placebo | 3.56 | 5.34 | 0.31 | 0.47 |
| ACT-DMD placebo | 3.25 | 4.87 | 0.40 | 0.60 |
| DEMAND III placebo | 2.91 | 4.36 | 0.32 | 0.47 |
| Drisapersen phase 2 placebo (NCT01153932) | 2.06 | 3.09 | 0.31 | 0.46 |
| By baseline 4SC completion time [velocity] |  |  |  |  |
| <2s [> 2 stairs/s] | 1.76 | 2.64 | 0.53 | 0.79 |
| 2 to 8s [0.5 to 2 stairs/s] | 2.38 | 3.57 | 0.31 | 0.46 |
| >8s [< 0.5 stairs/s] | 5.62 | 8.43 | 0.26 | 0.40 |
| By age group (years) |  |  |  |  |
| ≤7 | 2.20 | 3.30 | 0.37 | 0.55 |
| 7-12 | 3.19 | 4.78 | 0.35 | 0.52 |
| >12 | 3.79 | 5.69 | 0.36 | 0.53 |
