# Supplementary material for: Meaningful changes in motor function in Duchenne muscular dystrophy (DMD): A multi-center study
Source: PLoS One. 2024 Jul 10;19(7):e0304984. doi: 10.1371/journal.pone.0304984 (PMC11236155; doi:10.1371/journal.pone.0304984)
Supplement: Attachment — Submitted filename: Responses to reviewers-2-23-24.docx [file pone.0304984.s011.docx]

**Response to reviewer comments for PONE-D-23-24837**

**Reviewer #1

The study was conducted by the collaborative Trajectory Analysis Project (cTAP), a precompetitive coalition of academic clinicians, drug developers, and patient foundations formed in 2015 to overcome the challenges of high variation in clinical trials in DMD. They sought to determine:

1. Estimated minimal detectable change (MDC) for selected motor function measures in ambulatory DMD i.e., the minimal degree of measured change needed to be confident that true underlying change has occurred rather than transient variation or measurement error

2. Minimal clinically important differences (MCIDs) i.e., differences in function perceived as beneficial to patients

They selected outcome measures commonly used in clinical trials and clinical practice 6MWD, NSAA and 4SC and evaluated data collected across multiple clinical sites, networks and clinical trial placebo arms.

Main findings include the estimated MDCs and MCIDs for these outcome measures, the impact of baseline functional ability, and that there is generally very good consistency in the way these outcome measures are assessed and scored across sites with trained investigators both in the real world and as part of clinical trials.

This is an impressive analysis of a large set of available data. It is very nice to see placebo arm data from clinical trials being made available and used alongside clinical centre or natural history data. It provides assurance that with trained staff excellent consistency can be achieved in assessments performed at multiple sites across the world. It provides important benchmarks for future clinical trials.

I have no specific critique.**

**Response: Thank you for your review of our manuscript.**

**Reviewer #2

Muntoni et al. determined minimal detectable changes and minimal clinically important differences for motor function tests (North Star Ambulatory Assessment, 4-stair climb and 6-minute walk distance) in ambulant Duchenne muscular dystrophy patients using available cohort data from clinical trial arms, real-world data and natural history studies, that represent up to thousand patients and several thousand assessments. Results were sub-analyzed in respect to different cohort data, as well as age and state of disease progression at baseline. Results are thus representative for the overall DMD population. NSAA, 4-SC and 6MWD are widely used tests to determine motor function in DMD, and routinely used by many care centers across the world for the follow-up of patients, as well as being outcome measures in many clinical trials. Therefore, herein estimated MDC and MCID, as being applicable to individual patients, will improve patient follow-up and therapeutic decisions, as well as help to judge treatment benefits. The importance of the herein presented data cannot be overstated. I’m certain that herein presented data will be extremely well perceived by the community. From a personal view, herein calculated MDS and MCID match my clinical experience, what I want to say is that I’m not surprised by the values that make entirely sense, and I certainly will feel very comforted in future when using these values for e.g. judging disease progression.**

**I would like to congratulate authors to this important work, and I should say that it was a great honor that I could, as reviewer, be one of the first readers. The manuscript is written perfectly throughout. Data analysis, as far as I can judge (as lacking expertise in statistics), is carefully performed. The introduction is informative and the discussion is critical. I have no other general or specific comments or requests to the authors. Bravo!**

**Response: Thank you for your review of our manuscript and your kind comments.**

**Reviewer #3**

**Muntoni et al. conducted a study to examine the minimal detectable change (MDC) for NSAA, 4-SC, and 6MWD in ambulatory individuals with DMD. Overall, the study is well-designed with high methodological quality, and the manuscript is well-written and structured.

I only have a few comments. Please see below.

(1) In addition to MDC, the study also assessed anchor-based MCIDs for the motor function measures mentioned above. The importance of the MCID has been highlighted by clinicians, regulatory authorities, patients/parents, and their advocacy groups. Linking changes in an outcome measure to clinical meaningfulness for patients is of great significance to regulatory agencies involved in drug approval processes. However, the findings regarding MCIDs are not very reliable and meaningful in this study. I believe removing relevant part of MCIDs will not do any harm to the manuscript. Firstly, MCIDs were not one of the main objectives of this study. Secondly, there was limited data source available for analyzing MCIDs. Thirdly, as stated by the authors, functional motor scale (FMS) used in this study is not an appropriate anchor for determining MCIDs in DMD patients: FMS is not self-assessed by patients themselves, changes in FMS have weak correlations with changes in NSAA and 4SC, and increments in FMS represent too large of a difference in motor function.**

**Response: Thank you for this comment. We acknowledge the limitations of the FMS anchor and agree that it would be ideal to have a better-performing, patient-based measure as an anchor. However, we believe there is still value in including these results as a reference point for readers, while making the limitations clear, and noting the need for more sensitive estimation of MCIDs. We have stated the following in the manuscript to emphasize these points (lines 500-509):**

*“In fact the FMS, as used in the present study - where it was available in only one data source - should be rated as a low quality anchor for MCIDs in Duchenne [40]: FMS is not assessed directly by patients, changes in FMS have low correlations with changes in NSAA and 4SC, and the increments of FMS represent too large a difference in motor function. We think this makes the FMS a highly conservative anchor that most likely over-estimates MCIDs – i.e., smaller-than-MCID changes in NSAA and 4SC are likely meaningful to patients. More sensitive quantification of the clinical meaning associated with motor function changes in DMD is sorely needed to help decision-makers interpret the effects of novel therapeutics studied in clinical trials.”*

**We believe that including these results along with discussion of other anchor-based MCIDs in DMDs is valuable. We have included this in our discussion (lines 456-471):**

*“MCID* *estimates for 6MWD in DMD have been previously reported as ranging from 5.6 to 45.9 meters, depending on the level of baseline function [23], in a study of 24 boys in which the PODCI transfer and basic mobility scale was used as an anchor. Another study has reported MCIDs for the 4SC and other timed function tests using the Vignos scale as an anchor and a different statistical methodology (predicting changes in the anchor using logistic regression) [36]. The first two score levels of the Vignos scale are very similar to score levels 1 and 2 of the FMS, which was used as an anchor in the present study. The MCID for 4SC was estimated in this prior study as 0.035 tasks per second, corresponding to 0.14 stairs/second in the units of the present study and representing the degree of change in 4SC velocity required to predict a 1-unit change in the Vignos scale. The degree of accuracy in that prediction was low (AUC < 0.7) [36], consistent with the weak correlation between 4SC and FMS observed in the present study. These MCID estimates for 4SC are not directly comparable given differences in data and methodologies, but provide confidence that changes exceeding MDCs are meaningful to patients. An anchor-based MCID for the NSAA total score, anchoring on the 6MWD, has been estimated at 3.5 units [24].”*

**For the reasons above, we would prefer to retain this material in the manuscript.**

**(2) Page 9, line 193
Reference #4 was not relevant to the FMS. I haven't used it previously. I've looked for references on the psychometrics of FMS, but couldn't find any. Can you please provide relevant references regarding the FMS?**

**Thank you. We have corrected the reference, which should point to Swinyard et al. cited below. We also provide a juxtaposition of the FMS and better known Vignos scale below, which as seen below, are very similar for FMS levels 1 and 2.

Swinyard CA, Deaver GG, Greenspan l. Gradients of functional ability of importance in rehabilitation of patients with progressive muscular and neuromuscular diseases. Arch Phys Med Rehabil. 1957 Sep;38(9):574-9.
Vignos, P. J., Jr., et al. (1963). "Management of progressive muscular dystrophy in childhood." JAMA 184: 89-96.**

**Juxtaposition of the FMS and the Vignos scale**

| **Functional Motor Scale (Swinyard 1957)** | **Vignos Scale (1963)** |
| --- | --- |
| 1. Mild abnormalities in gait, able to climb stairs without assistance | 1. Walks and climbs stairs without assistance. |
| 2. More apparent gait abnormalities, requires a railing or support for stairs | 2. Walks and climbs stairs with aid of railing. |
| 3. Walks and arises from a chair independently, but cannot negotiate stairs without help | 3. Walks and climbs stairs slowly with aid of railing (over 25 sec for eight standard steps). |
| 4. Independent walking is the primary means of mobility, but a walker, braces or other means of support is necessary; unable to rise from a chair independently | 4. Walks unassisted and rises from chair but cannot climb stairs. |
| 5. Primary means of mobility is a wheelchair (WC), may stand with support for transfers; good trunk control in the chair and to perform all ADL from the chair | 5. Walks unassisted but cannot rise from chair or climb stairs. |
| 6. WC dependent and unable to stand, needs some help propelling a manual WC and with ADL | 6. Walks only with assistance or walks independently with long leg braces. |
| 7. Unable to propel a manual WC, some difficulty feeding self | 7. Walks in long leg braces but requires assistance for balance. |
| 8. Unable to sit without considerable support; requires maximal assistance for ADL | 8. Stands in long leg braces but unable to walk even with assistance. |
|  | 9. Is in wheel chair. Elbow flexors more than antigravity. |
|  | 10. Is in wheel chair or bed. Elbow flexors less  than antigravity. |

**Reviewer #4**

**This is a clinically meaningful research result that takes into account the use of RWD, which will become increasingly important in the future, and deserves to be adopted. There is nothing in particular to comment on.**

**Response: Thank you for your review of our manuscript.**

**Reviewer #5**

**This study tried to estimate the minimal detectable change (MDC) threshold > 80% confidence for selected motor function measures ( NSAA, 6MWT, 4SC) in ambulatory DMD, and the anchored based minimal clinically important differences (MCIDs) of NSAA or 4SC with 1 point worsening in FMS scores. The estimation is performed from the collected data of more than 1000 DMD ambulatory steroid treated patients , participating in either clinical trials placebo arms and real world clinical practice setting. It was found in this study that if an individual patient with DMD worsened in measured function by at least 3 units of the NSAA total score, or 40 meters of 6MWD,or 0.35 stairs/second for the 4SC, then in each case one can be at least 80% confident that he had experienced disease progression. The findings are useful for reference and the manuscript is well written.**

**After reading through the manuscript I have the following questions.

It was also mentioned that the study was trying to understand the meaningfulness of changes of these motor scales. It has been mentioned that such meaningfulness of changes can be estimated by following:

1. The minimal detectable change (MDC) threshold >80 % confidence or > 90% confidence for the selected motor function measures as a statistical estimated data as observed from a large number of DMD patients from clinical trials (placebo arms) and NH/RWD.
2. The meaningful changes to the families as in the changes in certain item in a certain motor measure.
3. The meaningful changes to the clinicians as using the FMS scores

While direct comparison of these different defined meaningful changes is not logical, what is the relationship of these different meaningful changes, and how these different meaningful dimensions supplementing each other worth further elaboration?**

**Response: Thank you for the comment. These three examples illustrate different conceptualizations of meaningfulness of changes.**

**The MDC, which is the focus of our study, is an estimate of the smallest magnitude of change attributable to transient biological or measurement variability; changes greater than this minimal threshold therefore likely reflect non-transient, underlying changes in function (lines 89-97).**

*“The present study estimates the minimal detectable change (MDC) in these measures of ambulatory function in DMD – that is, the minimal level of change in measured function that must be observed to be reasonably confident that true, underlying change in function has occurred for an individual (per item 1 above) rather than only transient changes (per items 2 and 3 above) [14, 15]. Understanding MDCs is important to the design and interpretation of clinical studies, and for monitoring real-world outcomes. Definitions of disease progression for use in clinical trial endpoints or treatment algorithms, for example, should be based on MDCs to ensure that signal for progression is distinguished from biological and measurement noise at the individual-patient level.”*

**The MDC is a statistical conceptualization of a meaningful change, which may differ from a patient’s or a caregiver’s view of a meaningful change. For example, as we noted in the discussion (lines 470-473), there is also evidence from patient and caregiver interviews that complete loss of function in a single NSAA item or deterioration in function of one or two items can be important to patients. Changes of this magnitude would fall under our MDC threshold of 2.8 points for the NSAA but may still be meaningful to patients or families.**

*“An anchor-based MCID for the NSAA total score, anchoring on the 6MWD, has been estimated at 3.5 units [24]. This study also conducted patient and caregiver interviews, which identified complete loss of function in a single NSAA item or deterioration of function in one to two items to be an important change.”*

**Similarly, differences in function assessed by a clinician (e.g., through changes in FMS score levels) represent another conceptualization of change, which, depending on the assessment, may capture changes that are smaller or greater than the MDC.**
 **Can the author also explain why the MDC was higher for boys with lower baseline 6MWD (60 meters among those with baseline 6MWT between 75 and 200 meters) than for boys with higher baseline 6MWD performance (~34 meters in boys with baseline 6MWT above 200 meters)?**

**Thank you for this question. We hypothesize that the higher estimated MDC for patients with lower baseline 6MWD may be due to greater variability in patients’ 6MWD performance as their function worsens to this level. The available data on 6MWD when patients reach these lower levels of function may also be less representative in real-world sources due to more non-assessment or missingness, as clinicians may be less likely to request tests such as the 6MWD that patients will find increasingly difficult because of their worsening function. Therefore, a larger MDC, may be required to be confident of true underlying change than at higher levels of function, where these issues are less of a concern. We have clarified this in the discussion (lines 397-401).**

*“Among patients starting from lower levels of function, slightly larger changes in NSAA, 6MWD and 4SC completion times were required to be confident that true change had occurred, suggesting that these assessments can have slightly more variability as function worsens. Non-assessment, and missing functional data, as boys approach loss of function could also contribute to this pattern.”*

**In the abstract it was also mentioned the identified thresholds can be used to inform endpoint definitions, as inputs into power calculations, or as benchmarks for monitoring individual and group-level changes in motor function in ambulatory DMD. Does the authors mean primary outcome definition and input into power calculation of primary end point for clinical trial, or otherwise ? What are the group-level changes for benchmarking? As the finding in the specific group level changes found in this study has not been mentioned in the abstract. Can the author also included the specific group level benchmarking findings in the result part ?**

**Thank you for the comment. Yes, the estimated MDC may be used to inform definition of trial endpoints and for benchmarking individual-level changes. For example, our estimated MDC of 2.8 units for NSAA total, may be considered a minimum threshold for a ‘progressor’ definition, such that any patient worsening beyond this threshold is considered to have a true worsening in function. Similarly, if a clinician is assessing a patient’s progression since their last clinical visit, a change of greater than 2.8 units on the NSAA indicates the patient’s changes are likely due to true disease progression, and are not explained by transient biological or measurement variability. We have clarified this in the *‘Applications to clinical trial and clinical practice’* section of the discussion (lines 476-481).**

*“An application of MDCs to clinical trials is under separate investigation by our group: a functional progression endpoint defined as the time from baseline to worsening function that exceeds the MDC. For example, our estimated MDC of 2.8 units for NSAA total, may be considered a minimum threshold for a ‘progressor’ definition, such that any patient worsening beyond this threshold is considered to have a true worsening in function.”*

**We would also like to clarify that power calculations should be based on MCIDs, rather than MDCs, which are a measure of meaningful individual-level change. Therefore, for clarity, we have removed mention of power calculations in the abstract. Similarly, we would also like to clarify that the MDC thresholds can be used as benchmarks for monitoring individual changes, and have removed mention of benchmarks for group-level changes (lines 69-72).**

*“The identified thresholds can be used to inform endpoint definitions, or as benchmarks for monitoring individual changes in motor function in ambulatory DMD.”*

**For ambulatory steroid treated DMD patients, most will loss ambulation before or at 16 years old. Yet the current age range is up to <18. What is the percentage of DMD patients in the current collective cohort is between 16-18 age?**

**Thank you for the comment. For the MDC analyses, patients were followed from the first time they meet all of the following criteria: 1) aged 4 to 18, 2) were on steroids, 3) had at least a minimal level of ambulatory function (as defined in the manuscript for each outcome, lines 210-214). Less than 1% of patients in the MDC analyses were age 16 or older at the start of follow-up. In our analyses of half SD, which used all available patient assessments in the pooled sample, only 2-4% of included assessments were from patients aged 16-18.**

**As such, the data summarized here is entirely from earlier ages and representative of the time frame when most DMD patients remain ambulatory.**

**From what I understand that the current study are on DMD patients in the placebo arm of clinical trials and in real clinical setting not receiving any disease modifying treatment. As it was mentioned that very few patients with 4SC and NSAA assessments over 48 weeks experienced an improvement in FMS, and such patients were excluded from the analysis. Is my understanding correct ? If so , can the authors have this clinical spelled out ?**

**Yes, this is correct. This applies to our anchor-based analysis, which used FMS as an anchor to estimate MCIDs for NSAA and 4SC. As there are very few patients improving from 2 to 1 on FMS who had NSAA and 4SC assessments, we did not have enough data to separately estimate MCIDs for improvement for these measures. We have revised the text in this section to be clearer (lines 239-247).**

*“Regression models were used to estimate the mean 48-week change in 4SC velocity or NSAA total score for patients with a 1-point worsening in FMS score (i.e., from 1 to 2, or from 2 to 3) versus those with no change in FMS over the 48-week period. Very few patients with 4SC and NSAA assessments over 48 weeks experienced a ˃1 point worsening. (Likewise, very few patients experienced an improvement in FMS (i.e., from 2 to 1) Given this small number of improving patients, we did not have sufficient data to estimate separate MCIDs for improvement.”*

**We have also clarified in other parts of the manuscript that what we are estimating for the MCID is an MCID for worsening (e.g., the abstract, lines 64, 120, 193, and 353).**

**Response to editorial comments for PONE-D-23-24837**

Journal requirements:

1. When submitting your revision, we need you to address these additional requirements.

Please ensure that your manuscript meets PLOS ONE's style requirements, including those for file naming. The PLOS ONE style templates can be found at 
[https://journals.plos.org/plosone/s/file?id=wjVg/PLOSOne_formatting_sample_main_body.pdf](https://nam10.safelinks.protection.outlook.com/?url=https%3A%2F%2Fjournals.plos.org%2Fplosone%2Fs%2Ffile%3Fid%3DwjVg%2FPLOSOne_formatting_sample_main_body.pdf&data=05%7C02%7CGautam.Sajeev%40analysisgroup.com%7Cc086e476a9ec4b955a3d08dc1de20daa%7C2be644011c664caea08292f4c2fca067%7C1%7C0%7C638418103951438256%7CUnknown%7CTWFpbGZsb3d8eyJWIjoiMC4wLjAwMDAiLCJQIjoiV2luMzIiLCJBTiI6Ik1haWwiLCJXVCI6Mn0%3D%7C3000%7C%7C%7C&sdata=%2B61ivy%2Bmru3ud6rrhUn0XeUHim%2BPiIx0OM8SWH%2FnpXo%3D&reserved=0) and 
[https://journals.plos.org/plosone/s/file?id=ba62/PLOSOne_formatting_sample_title_authors_affiliations.pdf](https://nam10.safelinks.protection.outlook.com/?url=https%3A%2F%2Fjournals.plos.org%2Fplosone%2Fs%2Ffile%3Fid%3Dba62%2FPLOSOne_formatting_sample_title_authors_affiliations.pdf&data=05%7C02%7CGautam.Sajeev%40analysisgroup.com%7Cc086e476a9ec4b955a3d08dc1de20daa%7C2be644011c664caea08292f4c2fca067%7C1%7C0%7C638418103951443060%7CUnknown%7CTWFpbGZsb3d8eyJWIjoiMC4wLjAwMDAiLCJQIjoiV2luMzIiLCJBTiI6Ik1haWwiLCJXVCI6Mn0%3D%7C3000%7C%7C%7C&sdata=wqeGsqe%2Bwb1wxh2N5j7s2QL%2Fgr835wQIWdRnfLmecDc%3D&reserved=0).

**Response: The additional formatting requirements have been followed.**

2. We note that the grant information you provided in the ‘Funding Information’ and ‘Financial Disclosure’ sections do not match.

When you resubmit, please ensure that you provide the correct grant numbers for the awards you received for your study in the ‘Funding Information’ section.

**Response: The funders in the two sections of the submission system are now identical.**

3. Thank you for stating the following in the Competing Interests section: 
[We have read the journal's policy and the authors of this manuscript have the following competing interests: 
Francesco Muntoni is a member of the Rare Disease Scientific Advisory Group for Pfizer and of Dyne Therapeutics SAB, and has participated to SAB meetings for PTC, Sarepta, Santhera, Wave Therapeutics. UCL and Great Ormond Street Hospital are recipient of grants from Pfizer, Italfarmaco, Wave, Santhera, Sarepta regarding clinical trials.
James Signorovitch co-founded the collaborative Trajectory Analysis Project (cTAP) and is an employee of Analysis Group, Inc., a consulting firm that received funding from the membership of cTAP to conduct this study.
Gautam Sajeev, Nicolae Done, and Henry Lane are employees of Analysis Group, Inc., a consulting firm that received funding from the membership of cTAP to conduct this study. Ibrahima Dieye and Zhiwen Yao were employees of Analysis Group, Inc. at the time this study was conducted. 
Nathalie Goemans has served on clinical steering committees and/or as a consultant and received compensation from Eli Lilly, Italfarmaco, PTC Therapeutics, and BioMarin Pharmaceutical; has served as site investigator for GlaxoSmithKline, Prosensa, BioMarin Pharmaceutical, Italfarmaco, Roche, and Eli Lilly.
Craig McDonald has served as a consultant for PTC Therapeutics, BioMarin Pharmaceutical, Sarepta Therapeutics, Eli Lilly, Pfizer Inc, Santhera Pharmaceuticals, Cardero Therapeutics, Inc, Catabasis Pharmaceuticals, Capricor Therapeutics, Astellas Pharma (Mitobridge), and FibroGen, Inc; serves on external advisory boards related to DMD for PTC Therapeutics, Sarepta Therapeutics, Santhera Pharmaceuticals, and Capricor Therapeutics; and reports grants from US Department of Education/National Institute on Disability and Rehabilitation Research, the National Institute on Disability, Independent Living, and Rehabilitation Research, US NIH/National Institute of Arthritis and Musculoskeletal and Skin Diseases, NIH/National Institute of Neurologic Disorders and Stroke, US Department of Defense, and Parent Project Muscular Dystrophy US.
Eugenio Mercuri has served on clinical steering committees and/or as a consultant for Eli Lilly, Italfarmaco, PTC Therapeutics, Sarepta, Santhera, and Pfizer; has served as PI for GlaxoSmithKline, Prosensa, BioMarin Pharmaceutical, Italfarmaco, Roche, PTC, Pfizer , Sarepta, Santhera, Wave , NS and Eli Lilly. EM reports personal fees as consultant, PI or member on advisory board form BIOGEN S.R.L. outside the submitted work; EM reports personal fees consultant, PI or member on advisory board from ROCHE; EM reports personal fees consultant, PI or member on advisory board form AVEXIS outside the submitted work; EM reports personal fees consultant, PI or member on advisory board from SCHOLAR ROCK outside the submitted work; EM is part of an institution that receives funding from Biogen for a SMA disease registry (ISMAR).
Erik H. Niks is a member of the European Reference Network for Rare Neuromuscular Diseases (ERN EURO‐NMD). EN report grants from Duchenne Parent Project, ZonMW and AFM, consultancies for BioMarin and Summit, and worked as local investigator of clinical trials of BioMarin, GSK, Lilly, Santhera, Givinostat, and Roche outside the submitted work. E.H.N. reports ad hoc consultancies for WAVE, Santhera, Regenxbio, and PTC, and he worked as investigator of clinical trials of Italfarmaco, NS Pharma, Reveragen, Roche, WAVE, and Sarepta outside the submitted work.
Brenda Wong has participated in advisory committee meetings for Prosensa and Biomarin and has received compensation for consultancy services for Gilead Sciences, Pfizer, GSK, RegenXBio, and PepGen.
Krista Vandenborne has received grants from NIH National Institute of Arthritis and Musculoskeletal and Skin Diseases/National Institute of Neurologic Disorders and Stroke, Parent Project Muscular Dystrophy, and the Muscular Dystrophy Association. She has also received funding from ltalfarmaco SpA, Sarepta Therapeutics, Summit Therapeutics plc, Catabasis Pharmaceuticals, Pfizer Inc, ldera Pharmaceuticals, Bristol-Myers Squibb, and Eli Lilly through grant awards to the University of Florida.
Volker Straub has participated in advisory boards for Audentes Therapeutics, Biogen, Exonics Therapeutics, Italfarmaco S.p.A., Roche, Sanofi Genzyme, Sarepta Therapeutics, Summit Therapeutics, UCB, and Wave Therapeutics. He has research collaborations with Ultragenyx and Sanofi Genzyme.
Imelda JM de Groot has no disclosures.
Cuixia Tian has participated as the site principal investigator for trials sponsored by PTC Therapeutics, Eli Lilly, GSK, Prosensa/Biomarin, Bristol Myers Squibb, Roche, Pfizer. Santhera, Sarepta, Fibrogen, Capricor, Pfizer, Avexis, and Catabasis.
Adnan Manzur has no disclosures.
Susan J. Ward co-founded and manages the collaborative Trajectory Analysis Project and has received funding from the membership of cTAP to facilitate this study.
Laurent Servais is member of the SAB or has performed consultancy for Sarepta, Dynacure, Santhera, Avexis, Biogen, Cytokinetics and Roche, Audentes Therapeutics and Affinia Therapeutics; LS has given lectures and has served as a consultant for Roche, Biogen, Avexis, and Cytokinetics. LS is the project leader of the newborn screening in Southern Belgium funded by Avexis, Roche, and Biogen.].

Please confirm that this does not alter your adherence to all PLOS ONE policies on sharing data and materials, by including the following statement: ""This does not alter our adherence to  PLOS ONE policies on sharing data and materials.” (as detailed online in our guide for authors [http://journals.plos.org/plosone/s/competing-interests](https://nam10.safelinks.protection.outlook.com/?url=http%3A%2F%2Fjournals.plos.org%2Fplosone%2Fs%2Fcompeting-interests&data=05%7C02%7CGautam.Sajeev%40analysisgroup.com%7Cc086e476a9ec4b955a3d08dc1de20daa%7C2be644011c664caea08292f4c2fca067%7C1%7C0%7C638418103951447801%7CUnknown%7CTWFpbGZsb3d8eyJWIjoiMC4wLjAwMDAiLCJQIjoiV2luMzIiLCJBTiI6Ik1haWwiLCJXVCI6Mn0%3D%7C3000%7C%7C%7C&sdata=UhtbAl6gb8U1a43YeUbw2C1kJZA76GClaT0%2BT9NoVNA%3D&reserved=0)).  If there are restrictions on sharing of data and/or materials, please state these. Please note that we cannot proceed with consideration of your article until this information has been declared.

Please include your updated Competing Interests statement in your cover letter; we will change the online submission form on your behalf.

**Response: We have included the updated the Competing Interests statement in the cover letter.**

4. In your Data Availability statement, you have not specified where the minimal data set underlying the results described in your manuscript can be found. PLOS defines a study's minimal data set as the underlying data used to reach the conclusions drawn in the manuscript and any additional data required to replicate the reported study findings in their entirety. All PLOS journals require that the minimal data set be made fully available. For more information about our data policy, please see [http://journals.plos.org/plosone/s/data-availability](https://nam10.safelinks.protection.outlook.com/?url=http%3A%2F%2Fjournals.plos.org%2Fplosone%2Fs%2Fdata-availability&data=05%7C02%7CGautam.Sajeev%40analysisgroup.com%7Cc086e476a9ec4b955a3d08dc1de20daa%7C2be644011c664caea08292f4c2fca067%7C1%7C0%7C638418103951452842%7CUnknown%7CTWFpbGZsb3d8eyJWIjoiMC4wLjAwMDAiLCJQIjoiV2luMzIiLCJBTiI6Ik1haWwiLCJXVCI6Mn0%3D%7C3000%7C%7C%7C&sdata=LLTu7yf0lfRHF1gYH6jHx4iBUQ3clfY0Z10InN1GQkw%3D&reserved=0).

""Upon re-submitting your revised manuscript, please upload your study’s minimal underlying data set as either Supporting Information files or to a stable, public repository and include the relevant URLs, DOIs, or accession numbers within your revised cover letter. For a list of acceptable repositories, please see [http://journals.plos.org/plosone/s/data-availability#loc-recommended-repositories](https://nam10.safelinks.protection.outlook.com/?url=http%3A%2F%2Fjournals.plos.org%2Fplosone%2Fs%2Fdata-availability%23loc-recommended-repositories&data=05%7C02%7CGautam.Sajeev%40analysisgroup.com%7Cc086e476a9ec4b955a3d08dc1de20daa%7C2be644011c664caea08292f4c2fca067%7C1%7C0%7C638418103951457724%7CUnknown%7CTWFpbGZsb3d8eyJWIjoiMC4wLjAwMDAiLCJQIjoiV2luMzIiLCJBTiI6Ik1haWwiLCJXVCI6Mn0%3D%7C3000%7C%7C%7C&sdata=kaQcztDQe%2F2wL3JnziuJ1FV6G7QaRus651eLhrsr4I0%3D&reserved=0). Any potentially identifying patient information must be fully anonymized.

Important: If there are ethical or legal restrictions to sharing your data publicly, please explain these restrictions in detail. Please see our guidelines for more information on what we consider unacceptable restrictions to publicly sharing data: [http://journals.plos.org/plosone/s/data-availability#loc-unacceptable-data-access-restrictions](https://nam10.safelinks.protection.outlook.com/?url=http%3A%2F%2Fjournals.plos.org%2Fplosone%2Fs%2Fdata-availability%23loc-unacceptable-data-access-restrictions&data=05%7C02%7CGautam.Sajeev%40analysisgroup.com%7Cc086e476a9ec4b955a3d08dc1de20daa%7C2be644011c664caea08292f4c2fca067%7C1%7C0%7C638418103951462782%7CUnknown%7CTWFpbGZsb3d8eyJWIjoiMC4wLjAwMDAiLCJQIjoiV2luMzIiLCJBTiI6Ik1haWwiLCJXVCI6Mn0%3D%7C3000%7C%7C%7C&sdata=j%2BZStQWdyJvmf1h%2BMivCx%2FHvNLI4tu333iI21GwlNh8%3D&reserved=0). Note that it is not acceptable for the authors to be the sole named individuals responsible for ensuring data access.

We will update your Data Availability statement to reflect the information you provide in your cover letter.

**Response: We have included the updated the Data Availability statement in the cover letter.**

5. One of the noted authors is a group [Association Française contre les Myopathies, The UK NorthStar Clinical Network, ImagingDMD investigators, cTAP]. In addition to naming the author group, please list the individual authors and affiliations within this group in the acknowledgments section of your manuscript. Please also indicate clearly a lead author for this group along with a contact email address.

**Response: We have listed the member names for all author groups and indicated the lead author for each group along with their email address.**

6. Please include captions for your Supporting Information files at the end of your manuscript, and update any in-text citations to match accordingly. Please see our Supporting Information guidelines for more information: [http://journals.plos.org/plosone/s/supporting-information](https://nam10.safelinks.protection.outlook.com/?url=http%3A%2F%2Fjournals.plos.org%2Fplosone%2Fs%2Fsupporting-information&data=05%7C02%7CGautam.Sajeev%40analysisgroup.com%7Cc086e476a9ec4b955a3d08dc1de20daa%7C2be644011c664caea08292f4c2fca067%7C1%7C0%7C638418103951467962%7CUnknown%7CTWFpbGZsb3d8eyJWIjoiMC4wLjAwMDAiLCJQIjoiV2luMzIiLCJBTiI6Ik1haWwiLCJXVCI6Mn0%3D%7C3000%7C%7C%7C&sdata=9mejXUefKOMmCONFA%2BMpNYkNxytrsw2vt4yshmkV0pE%3D&reserved=0).

**Response: Captions for the Supporting Information files are now included at the end of the manuscript.**

7. Please review your reference list to ensure that it is complete and correct. If you have cited papers that have been retracted, please include the rationale for doing so in the manuscript text, or remove these references and replace them with relevant current references. Any changes to the reference list should be mentioned in the rebuttal letter that accompanies your revised manuscript. If you need to cite a retracted article, indicate the article’s retracted status in the References list and also include a citation and full reference for the retraction notice.

**Response: Reference list has been reviewed, and any changes are indicated in the response letter.**
