# Supplementary material for: Meaningful changes in motor function in Duchenne muscular dystrophy (DMD): A multi-center study
Source: PLoS One. 2024 Jul 10;19(7):e0304984. doi: 10.1371/journal.pone.0304984 (PMC11236155; doi:10.1371/journal.pone.0304984)
Supplement: Attachment — Submitted filename: Responses to Reviewers - 5-13-24.docx [file pone.0304984.s012.docx]

**Response to reviewer comments for PONE-D-23-24837R1**

**Reviewer #2**

I was already happy with the first version, now the manuscript improved further following reviewing process. Perfect manuscript.

**Response: We thank the Reviewer for the kind words.**

**Reviewer #3**

All my concerns have been well addressed. Great work.

**Response: We thank the Reviewer for the kind words.**

**Reviewer #4**

All comments from reviewers have been answered appropriately and there are no additional points to comment.

**Response: We thank the Reviewer for taking the time to review our work.**

**Reviewer #5**

Thank you for the thoroughness in explanation and have the relevant changes incorporated in the updated manuscript. The current manuscript with the updated explanation and modifications will help the readers to understand better the significance of the current findings and the clinical interpretation when applying to clinical setting. I have no further comment. Congratulation to the team for this extensive and important work.

**Response: We thank the Reviewer for the positive comments.**

**Reviewer #6**

The authors intended to evaluate the minimal clinically important differences to determine the minimal degree of measured change needed with > 1000 Duchenne muscular dystrophy (DMD) patients. They reported the identified thresholds for monitoring individual changes in motor function in ambulatory DMD.

1. Line 118. The patient samples were from various resources. Is there any potential heterogeneity to be concerned?

**Response: Thank you for this question. We discuss consistency of our estimates of minimal detectable change (MDC) across data sources used in our analysis in lines 354-381 of the manuscript, and report these results in the supplementary tables (S6 – S9 Tables).**

**To summarize, we obtained similar results for NSAA total score, 6MWD, and 4SC velocity across data sources, with differences among individual data sources being small relative to the size of the MDCs. Our MDC estimates (with 80% confidence) were 2.78 for NSAA total (ranging from 2.68 to 3.16 across data sources), 36.3 meters for 6MWD (30.5 to 41.5 meters across data sources), and 0.35 for 4SC velocity (0.26 to 0.42 or 0.27 to 0.44 across data sources, depending on the value used for truncation of 4SC completion times). There was more heterogeneity in MDCs for 4SC completion time, which varied based on the truncation time: 1.30 seconds when completion times were truncated at 12 seconds (1.05 to 1.90 seconds across data sources) and 3.21 seconds when completion times were truncated at 30 seconds (1.81 to 4.97 seconds across data sources).**

**Overall, these results suggest high consistency in MDCs for NSAA total score, 6MWD, and 4SC velocity across sources. There was more heterogeneity in estimates of MDCs for 4SC completion times. This could be mitigated by having consistent protocols for conducting and recording of 4SC that standardize: a) how long an unassisted attempt is allowed to go on before stopping the test, and b) how to document inability or the decision not to conduct a test. These points as well as likely reasons for the consistency in 6MWD and NSAA total score are discussed in lines 354-381 of the manuscript.**

2. Line 134. “Clinical co-authors of this manuscript who cared for patients at the included RWD/NHD sources may have access to information that could identify individual participants during or after data collection.” Will their access have a potential effect on sample selection or data collection?

**Response: No. This note just reflects that several of the co-authors are PIs at the centers from which data were used for this study. All eligible data from patients at these centers was used in this analysis. Data collection in each source was based on a specific protocol or via assessments conducted as part of patients’ routine follow-up care.**

3. Line 174. “In RWD sources, …… may not require a patient with very poor function to attempt the test….”. This type of missing data would easily introduce bias results to be evaluated.

**Response: We agree that this is a limitation of 4SC data collection in the real-world/natural history data sources. To address this, we explored different truncation thresholds for 4SC completion times and also looked at 4SC velocities. As noted above and discussed in lines 366-381, we found that 4SC velocity results are robust to differences in test conduct across care settings, while MDC estimates based on 4SC completion times are less consistent, and varied by truncation threshold.**

**Response to editorial comments for PONE-D-23-24837R1**

Journal requirements:

Please review your reference list to ensure that it is complete and correct. If you have cited papers that have been retracted, please include the rationale for doing so in the manuscript text, or remove these references and replace them with relevant current references. Any changes to the reference list should be mentioned in the rebuttal letter that accompanies your revised manuscript. If you need to cite a retracted article, indicate the article’s retracted status in the References list and also include a citation and full reference for the retraction notice.

**Response: The reference list has been reviewed, and no changes were made.**

Additional Editor Comments:

Please respond to the following reviewer's comments:

The authors intended to evaluate the minimal clinically important differences to determine the minimal degree of measured change needed with > 1000 Duchenne muscular dystrophy (DMD) patients. They reported the identified thresholds for monitoring individual changes in motor function in ambulatory DMD.

1. Line 118. The patient samples were from various resources. Is there any potential heterogeneity to be concerned?

2. Line 134. “Clinical co-authors of this manuscript who cared for patients at the included RWD/NHD sources may have access to information that could identify individual participants during or after data collection.” Will their access have a potential effect on sample selection or data collection?

3. Line 174. “In RWD sources, …… may not require a patient with very poor function to attempt the test….”. This type of missing data would easily introduce bias results to be evaluated.

**Response: We have responded to each of the above Reviewer comments. Specifically, please see our responses to Reviewer #6.**
